# Neurodevelopmental impairments in children with septo-optic dysplasia spectrum conditions: a systematic review

**DOI:** 10.1186/s13229-023-00559-0

**Published:** 2023-07-25

**Authors:** Amy Mann, Arameh Aghababaie, Jennifer Kalitsi, Daniel Martins, Yannis Paloyelis, Ritika R. Kapoor

**Affiliations:** 1 Department of Neuroimaging, Institute of Psychiatry, Psychology and Neuroscience, King’s College London, London, UK; 2 Homerton Healthcare NHS Trust, Homerton University Hospital, London, UK; 3 Florence Nightingale Faculty of Nursing, Midwifery and Palliative Care, Child and Family Health Nursing, King’s College London, London, UK; 4 NIHR Maudsley Biomedical Research Centre, South London and Maudsley NHS Trust, London, UK; 5 Department of Paediatric Endocrinology, Variety Children’s Hospital, King’s College Hospital NHS Foundation Trust, London, UK; 6 Faculty of Life Sciences and Medicine, King’s College London, London, UK

**Keywords:** Septo-optic dysplasia, Optic nerve hypoplasia, Autism spectrum disorder, Neurodevelopmental impairments, Neurobehavioural impairments

## Abstract

**Background:**

Septo-optic dysplasia (SOD) is a rare condition diagnosed in children with two or more of the following: hypopituitarism, midline brain abnormalities, and optic nerve hypoplasia. Children with SOD experience varied visual impairment and endocrine dysfunction. Autistic-like behaviours have been reported; however, their nature and prevalence remain to be fully understood. The present systematic review aimed to explore the type and prevalence of neurodevelopmental impairments in children with SOD spectrum conditions.

**Methods:**

The search was conducted in PubMed, EMBASE, and PsycInfo. Hand-searching reference lists of included studies was conducted. All peer-reviewed, observational studies assessing behavioural and cognitive impairments or autism spectrum disorder (ASD) symptoms in children (< 18 years) with SOD, optic nerve hypoplasia, and SOD-plus were included. Studies were excluded if they did not report standardised measures of neurodevelopmental impairments or ASD outcomes.

**Results:**

From 2132 screened articles, 20 articles reporting data from a total of 479 children were included in prevalence estimates. Of 14 studies assessing cognitive-developmental outcomes, 175 of 336 (52%) children presented with intellectual disability or developmental delay. A diagnosis of ASD or clinical level of symptoms was observed in 65 of 187 (35%) children across five studies. Only five studies assessed for dysfunction across behavioural, emotional, or social domains and reported impairments in 88 of 184 (48%) of children assessed.

**Limitations:**

Importantly, high heterogeneity among the samples in relation to their neuroanatomical, endocrine, and optic nerve involvement meant that it was not possible to statistically assess the relative contribution of these confounding factors to the specific neurodevelopmental phenotype. This was further limited by the variation in study designs and behavioural assessments used across the included studies, which may have increased the risk of information bias.

**Conclusions:**

This systematic review suggests that the prevalence of neurodevelopmental impairments in children within the SOD spectrum may be high. Clinicians should therefore consider including formal assessments of ASD symptoms and neurodevelopmental impairments alongside routine care. There is, additionally, a need for further research to define and validate a standardised battery of tools that accurately identify neurodevelopmental impairments in SOD spectrum conditions, and for research to identify the likely causal mechanisms.

**Supplementary Information:**

The online version contains supplementary material available at 10.1186/s13229-023-00559-0.

## Background

Septo-optic dysplasia (SOD) is a rare congenital condition diagnosed in children with two or more of: pituitary hormone abnormalities, optic nerve hypoplasia (ONH, i.e. underdevelopment of the optic nerve), and midline brain abnormalities (e.g. agenesis of the corpus callosum) [1]. Males and females are equally affected, with an incidence of 1/10000 [1]. Children with SOD have a heterogeneous clinical phenotype with varying severity of abnormalities, including delayed cognitive development, endocrine dysfunction, sleep disturbance, and visual impairment. Emerging reports of neurodevelopmental impairments characteristic of autism spectrum disorder (ASD) have increasingly been reported in children with SOD. Despite having considerable impact on quality of life, the nature of these impairments remains to be fully understood. Consequently, this lack of understanding, in combination with medical prioritisation of somatic features of the condition, means that behavioural disturbances and daily-life disabilities are frequently overlooked and not considered in routine care.

Current understanding of the neurobehavioural phenotype of SOD is largely limited by the heterogeneity of its clinical phenotype. Recent discussions have argued re-defining SOD as a spectrum-based condition to encapsulate all variations of its clinical presentation [2]. For example, ONH is likely to represent one end of the SOD spectrum as it often presents with additional hypothalamic dysfunction. Moreover, patients with ONH may go on to develop pituitary hormone deficiencies [3]. SOD can also present with cortical development malformations, such as schizencephaly, diagnosed as SOD-plus [4]. In most cases, children with SOD-plus exhibit severe cognitive impairment, seizures, and visual impairment [4]. Approaching SOD as a complex spectrum-based condition requiring a holistic assessment of neurodevelopmental impairments in addition to somatic symptoms should therefore be considered. Taking into account the specific clinical features may allow for a more precise characterisation of the neurobehavioural phenotype.

Reports of neurodevelopmental impairments in SOD spectrum conditions are primarily centred around developmental delay, ranging from observations of isolated deficits (i.e. motor impairment) to global delays [5]. Neuropsychiatric symptoms [6] and behaviours characteristic of attention deficit hyperactivity disorder (ADHD) [7] have further been noted. In recent years, accumulating reports of ASD-like features in children with SOD spectrum conditions have emerged suggesting ASD as a possible association. ASD is a neurodevelopmental disorder diagnosed by deficits in social communication, and restrictive and repetitive behaviours [8]. Difficulties in social communication, echolalia (i.e. repetition of others speech), stereotyped behaviours, and deficits in peer interactions have been noted in children with SOD spectrum [9]. However, many of these observations are limited to opinion papers and case reports, often formulated in the absence of validated tools that would be important to objectively identify the presence and severity of neurodevelopmental impairments.

There are additional complexities regarding the assessment of ASD-like features in children with SOD spectrum conditions related to the common presence of visual impairment. First, tools that have been validated for the assessment of neurodevelopmental impairments and neuropsychiatric symptoms in sighted populations may not be suitable for populations with visual impairment. For example, their use may result in the overestimation of social cognitive deficits [10]. An additional concern is whether ASD-like behaviours in children with visual impairment represent secondary, blind-specific abnormalities in the development of social cognition, as opposed to primary neurodevelopmental deficits [11]. For example, a visually impaired child may use echolalia to aid language development rather than as a self-expression response as seen in ASD; limited exposure to visual social cues during early development may subsequently give rise to social communication abnormalities [12].

The present systematic review aims to assess the type and prevalence of neurodevelopmental impairments and ASD symptoms in children with SOD spectrum conditions. At present, neurodevelopmental impairments are not routinely assessed and therefore remain untreated. It is only when caregivers raise concerns about experiencing difficulty with managing their child’s behaviour at home and during school that these aspects of the condition are addressed by clinicians. It is anticipated that by improving understanding of the neurodevelopmental impairments and their aetiology in children with SOD spectrum conditions, early intervention and treatment plans may be developed to aid symptom management and improve patient and caregiver quality of life. It should be noted that for the purpose of this review, the term neurodevelopmental impairments will be used to encapsulate all behavioural, social, emotional, and cognitive-developmental impairments.

Case example A 13-year-old female patient diagnosed with septo-optic dysplasia was seen in paediatric endocrine clinics for multiple pituitary hormone deficiencies. She is treated with hydrocortisone, levothyroxine, and desmopressin acetate, and also received treatment for precocious puberty. She additionally has severe visual impairment. Alongside the somatic features of the condition, she presents with behaviourally induced insufficient sleep syndrome and autistic-like behaviours. Specifically, she experiences heightened auditory sensitivities that severely impact her everyday life including her education, self-care, play, and social interactions. These behavioural difficulties were only addressed when raised by her caregivers. Adjustments have now been put in place so that she attends clinic wearing ear defenders and the clinicians speak in soft tones to help her cope with her auditory sensitivities and minimise distress during clinic visits.

## Methods

### Search strategy

This systematic review followed the PRISMA guidelines to identify relevant literature assessing neurodevelopmental impairments in children with septo-optic dysplasia (SOD) spectrum conditions. A protocol was developed a priori and registered with the Open Science Framework (https://doi.org/10.17605/OSF.IO/45R9A). The search was performed in PubMed and OVID databases, EMBASE and PsycInfo of peer-reviewed journal articles published in English, from inception through July 16, 2022. These databases were used due to their relevance to the target field. The following search terms were used: (septo-optic dysplasia OR septo-optic dysplasia OR septo-optic dysplasia OR ONH OR optic nerve hypoplasia) AND (neurobehav* OR behav* OR social* OR emotion* OR affect OR affective OR problem* OR cognit* OR psych* OR develop* OR neurodevelop*). Search terms were truncated as appropriate to maximise retrieval. No filters or limits were applied to the search. Additional sources of literature included hand-searching reference lists of included studies and relevant review papers.

### Study selection

Duplicated articles were removed using the Rayyan duplicate identification tool. Following this, all authors independently reviewed the titles and abstracts against the inclusion and exclusion criteria, ensuring that each reference was independently screened by two reviewers. Full-text articles identified for further scrutiny were independently reviewed by all authors for eligibility, as per described above. Discrepancies on inclusion of an article were discussed and reviewed by a fourth reviewer (RRK) to maintain reliability throughout the study selection process. Given the nature of this systematic review, our inclusion criteria were intentionally broad to minimise search bias and comprised of original peer-reviewed articles with: (1) a sample of children up to 18 years old with SOD, SOD-plus, or ONH; and (2) a formal assessment of neurodevelopmental (behavioural, cognitive-developmental, social, or emotional) impairments or autism spectrum disorder symptoms using validated measures, such as the Social Communication Questionnaire (SCQ) [13], the Child Behaviour Checklist (CBCL) [14] or diagnostic tools (e.g. DSM-V) [8]. Due to discrepancies in the diagnostic classification of SOD, grey literature was not included as this may have limited our ability to comprehensively appraise the study results by condition subgroup. Non-English articles and those where full-texts were not available were excluded due to the inability to extract required study information and data. All excluded articles were documented with justifications in Excel.

### Data charting

A data extraction spreadsheet was drafted in Excel during protocol development. Data extraction was trialled in the first five references and adjustments were made to ensure all relevant study information was obtained. Categories in the data extraction spreadsheet included: (1) first author, (2) year of publication, (3) study design and sample size, (4) age and sex of participants, (5) SOD condition type, (6) severity of visual impairment, (7) comparator group demographics (if applicable; see Additional File 1), (8) outcome measures used, (9) prevalence statistics and/or mean outcome results, (10) ASD diagnosis (if applicable), and (11) endocrine deficiencies and/or neuroimaging results (if reported). Using the data extraction tool, AM and AA/JK independently extracted the data from each study. Where SOD, ONH, and SOD-plus participants were included within a sample of children with other conditions (e.g. isolated septal agenesis), only data for the target populations were extracted. A critical appraisal tool was not used to assess the quality of the included studies due to high variation among the study designs. Information on demographic and clinical data are reported in Table 1. A narrative synthesis approach was used to integrate the key findings of the included studies.

**Table 1:** Demographic and clinical data of included studies

First Author	Study design	Sample size (*N*)^a^	Reported condition^b^	Sex (*N*)	Age range (years)	Optic nerve involvement (%)	Endocrine dysfunction (%)	Neuroimaging abnormalities (%)
Webb, [15]	Prospective	11	Isolated ONH	2 F, 9 M	1–11	7/11 (64) bilateral ONH, 4/11 (36) unilateral ONH 11/11 (100) normal to mild/moderate VI	0/11 endocrine dysfunction	0/11 midline brain abnormalities
Dahl, [16]	Population-based cross-sectional cohort	65	ONH	33 F, 32 M	6.1–25.5^c^	35/65 (54) bilateral ONH: 12/35 BCVA < 0.05 (blind), 1/35 BCVA 0.05—< 0.1 severe VI, 5/35 BCVA > 0.1 moderate VI, 17/35 mild to no VI 30/65 (46) unilateral ONH: 29/29 mild to no VI	–	–
Ek, [30]	Retrospective cohort	13	ONH	7 F, 6 M	4.5–13^d^	13/13 (100) bilateral ONH All severe VI^e^	11/13 (85) hormonal deficiencies (GHD, TSH, ACTH, ADH, prolactin)	11/13 (85) midline brain malformations, 9/13 (69) absent SP, 7/13 (54) pituitary abnormalities
Fahnehjelm, [17]	Retrospective cohort	28	ONH	–	4.0–9.2^d^	28/28 bilateral ONH^e^	16/28 (57) hormonal deficiencies (e.g. GHD, multiple pituitary hormone deficiencies)	12/22 (55) small/absent/ectopic PP, 4/21 small AP, 14/26 (54) absent SP or CC, 9/26 (35) other cerebral findings
Fahnehjelm, [20]	Population-based cross-sectional	66	ONH	34 F, 32 M	0.6–19.4 ^d, f^	22/40 (55) bilateral ONH, 18/40 (45) unilateral ONH	–	–
Garcia-Filion, [21]	Prospective	73	ONH	31 F, 42 M	5 (age at neurobehavioural assessment)	60/73 (82) bilateral ONH, 13/73 (18) unilateral ONH	57/72 (79) endocrine dysfunction (e.g. GHD, hypothyroidism)	25/65 (38) CCH, 25/65 (38) absent SP, 8/63 (13) pituitary gland abnormality, 9/65 (14) other malformations (e.g. schizencephaly)
Groenveld, [23]	Cohort	19	ONH	10 F, 9 M	5–14.5	19/19 (100) bilateral ONH 5/19 (26) near-total blindness, 7/19 (37) severe-profound VI, 7/19 (37) moderate VI	3/13 (23) intact SP endocrine dysfunction No endocrine dysfunction in absent SP group	6/19 (32) absent SP, 13/19 (68) intact SP
Margalith, [6]	Retrospective cohort	51	Congenital ONH	25 F, 26 M	Up to 15^ g^	47/51 (92) bilateral ONH, 4/51 (8) unilateral ONH Each eye separately: 11/102 (11) no light perception, 31/102 (30) light perception only, 51/102 (50) partial vision, 10/102 (10) normal acuity	19/21 (90) endocrine dysfunction	34/38 (90) neuroradiological abnormalities e.g. 13/26 (50) absent SP, 3/38 (8) agenesis of CC, 1/38 (3) schizencephaly
Rivkees, [24]	Prospective	19	ONH	–	2 < ^h^	16/19 (84) bilateral ONH	11/19 (58) GHD, 6/19 (32) THD, 8/19 (42) AHD, 2/19 (11) DI	8/19 (42) CCH
Williams, [28]	Cross-sectional	7	ONH	3 F, 4 M	3.75–19.2	4/7 (57) bilateral ONH, 3/7 (43) unilateral ONH	0/7 endocrine dysfunction	7/7 (100) absent SP; 3/7 (43) thinning of corpus callosum
Williams, [29]	Cross-sectional	7	ONH	4 F, 3 M	5–9	2/7 (29) no light perception, 2/7 (29) light perception only, 3/7 (43) light perception in one eye	5/6 (83) endocrine dysfunction	3/7 (43) structural brain abnormality (e.g. CCH, absent SP)
Jutley-Neilson, [32]	Cross-sectional	41	ONH ‘spectrum’	18 F, 23 M	3–16	15/41 (37) mild-moderate VI, 12/41 (29) severe-profound VI, 14/41 (34) near-total visual loss	–	–
Jutley-Neilson, [31]	Cross-sectional	42	28 SOD, 14 ONH	18 F, 24 M	3–16	16/42 (38) mild-moderate VI, 12/42 (29) severe-profound VI, 14/42 (33) total vision loss	–	–
Parr, [33]	Retrospective	83	55 SOD, 28 ONH	45 F, 38 M	0.8–6.8	42/83 (51) profound VI, 41/83 (49) severe VI	–	28/83 (34) additional CNS abnormality (e.g. underdevelopment of pons, cerebral malformations, hydrocephalus, white matter abnormalities, and schizencephaly)
Griffiths, [22]	Case report	1	SOD	F	13.3	Bilateral ONH	Subnormal levels of GH, ACTH, cortisol deficiency	Absent SP
Severino, [18]	Retrospective case–control	38	SOD	17 F, 21 M	2 days–18.3^i^	27/38 (71) ONH	10/38 (26) endocrine dysfunction (e.g. GHD, TSH, ACTH deficiency)	21/38 (55) midbrain-hindbrain abnormalities, 14/38 (37) cortical malformations, 21/38 (55) brain stem abnormalities, 26/38 (68) pituitary abnormalities
Vawter-Lee, [26]	Retrospective	2	SOD	1 F, 1 M	0.7–1.75	1/2 (50) bilateral ONH	1/2 (50) adrenal insufficiency	2/2 (100) absent SP
Webb, [27]	Cross-sectional	6	SOD	2 F, 4 M	1–7	4/6 (67) bilateral ONH, 2/6 (33) unilateral ONH 2/6 (33) profound VI, 4/6 (67) severe VI	6/6 (100) endocrine deficiencies (e.g. GHD, ACTH, TSH)	6/6 (100) abnormalities (e.g. absent SP, small AP, thin CC)
Alt, [19]	Retrospective	17	1 SOD, 3 SOD-like, 13 SOD-plus	8 F, 9 M		16/17 (94) bilateral ONH^j^ 17/17 (100) poor vision/blindness	11/17 (65) endocrine deficiencies (e.g. GHD, TSH)	13/17 (76) midline defect, 11/17 (65) SP agenesis, 2/17 (12) hypogenesis of CC, abnormal HP axis 9/17 (53), cortical malformations 13/17 (76)
Signorini, [25]	Retrospective	17	7 SOD, 3 SOD-like, 7 SOD-plus	8 F, 9 M	0.3–9.5	16/17 (94) bilateral ONH, 1/17 (6) unilateral ONH 7/17 (41) light perception only, 2/17 (12) very low vision, 7/17 (41) low vision, 1/17 (6) near normal	9/17 (53) endocrine deficiencies (all multiple pituitary hormone deficiencies)	14/17 (82) midline brain defects, 7/17 (41) cortical developmental malformations

*–* Indicates not assessed, *NR* Not reported, *BCVA* Best corrected visual acuity, *VI* Visual impairment, *GHD* Growth hormone deficiency, *TSH* Thyroid stimulating hormone, *ACTH* Adrenocorticotropic hormone, *ADH* Antidiuretic hormone, *SP* Septum pellucidum, *PP* Posterior pituitary gland, *AP* Anterior pituitary gland, *CC* Corpus callosum, *THD* Thyroid hormone deficiency, *AHD* Adrenal hormone deficiency, *DI* Diabetes insipidus, *CCH* Corpus callosum hypoplasia, *HP* Hypothalamo-pituitary

^a^Sample size of target populations (ONH, SOD, SOD-plus) reported

^b^Condition reported by the original authors has been detailed in the table, please refer to endocrine and neuroimaging columns

^c^Age range reported for time of analysis, not age at assessment; median age 14.1 years

^d^Age range at time of neurobehavioural assessment

^e^Only children with severe visual impairment referred to centre used for recruitment were included

^f^Children in clinical analysis < 18 years (*n *= 40)

^g^One participant 23 years of age at assessment

^h^Children aged 2 years and older; mean age 3.6 years

^i^Age range reported at time of scan; not age at developmental assessment

^j^Diagnosis using MRI—14 bilateral and 3 unilateral from clinical examination

## Results

**Fig. 1:**
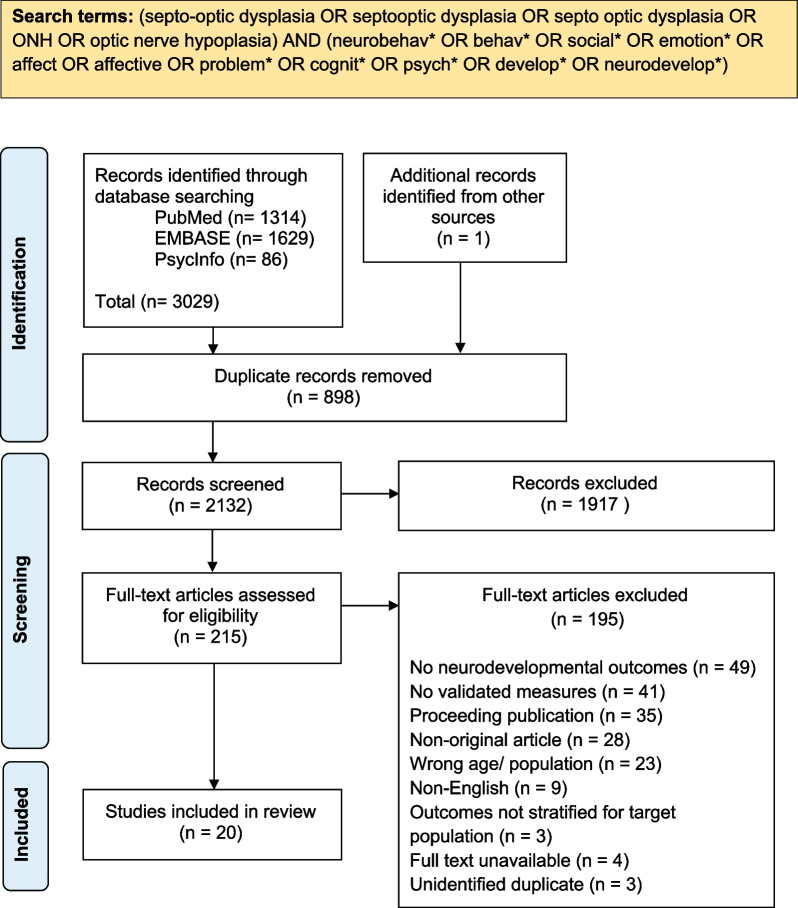
PRISMA flowchart of search strategy

Figure 1 details the study retrieval, selection, and inclusion results in a PRISMA flow diagram. In total, the search resulted in 2132 unique records. Two hundred and fifteen articles were assessed for eligibility. Following full-text review against the inclusion and exclusion criteria, 20 articles were included.

### Characteristics of included studies

Eight of the twenty studies reported SOD/SOD-plus patients within their study samples; the majority of studies (*N *= 12) reported samples of only patients with ONH. However, the diagnoses of all but one [15] of these 12 studies may be contested either due to reports of endocrine deficiencies and neuroanatomical abnormalities in their samples, which suggests they met the criteria for SOD (*N *= 8), or due to the lack of such reports (*N *= 3; see Table 1). For example, Dahl et al. [16] classified their sample as ONH; however, no endocrine or neuroimaging data were reported; therefore, it is unknown whether these children experienced the additional clinical features of SOD.

Optic nerve involvement was reported across all included studies. These reports were varied, with one study reporting the proportion of participants with ONH as a feature of SOD [18]. Some studies identified the proportion of bilateral (affecting both branches of the optic nerve) and unilateral ONH within their sample [6, 15–17, 19–28, 30,] and other studies reported severity of visual impairment using: best corrected visual acuity (BCVA) [16]; visual acuity in relation to level of light perception (i.e. no light perception, light perception only) [6, 25, 29]; or visual impairment as mild-moderate, severe-profound, or near-to-total visual loss [15, 23, 27, 30–33].

Varied tools were used to assess neurodevelopmental impairments and ASD outcomes across the 20 included studies reported in Table 2. Most studies (14 of 20 studies) assessed cognitive-developmental outcomes, including intellectual disability and developmental delay. Intellectual disability was defined as an intelligence quotient of less than 70. The assessment of developmental outcomes varied across the studies but mainly included social-emotional, adaptive (i.e. personal self-help behaviours), motor, communication, and cognitive domains. Some authors assessed the presence of global developmental delay as a binary score of delayed versus not delayed; a developmental quotient of less than 70 was commonly used to indicate delay. Only five studies formally assessed behavioural, emotional, or social outcomes [15, 28, 31–33], and five studies provided a formal assessment of ASD [16, 29–31, 33]. The same sample was used for Jutley-Neilson et al. [31] and Jutley-Neilson et al. [32]; therefore, only unique results are discussed. Below, we summarise the main results from each of these three domains in turn.

**Table 2:** Prevalence of cognitive-developmental; behavioural, social, or emotional, and ASD in 20 included studies

First Author	Reported condition(s)	Prevalence *N* (%)			Measures used
		Cognitive-developmental Impairment	Behavioural, emotional, social impairment	ASD diagnosis/cut-off	
Webb, [15]	Isolated ONH	–	4/11 (36) CBCL	–	CBCL
Dahl, [16]	ONH	20/55 (36)	–	7/42 (17)	FTF, WISC-IV, WAIS-IV, WPPSI, DSM-IV, DSM-V
Ek, [30]	ONH	5/13 (38)	–	6/13 (46)^b^	WISC-III, WPPSI-R, GDS, NEPSY, RZS, ITVIC. DSM-IV, ADI-R, CARS-3
Fahnehjelm, [17]	ONH	9/24 (38)	–	*8/28 (29)*^*c*^	WISC-III, WPPSI, GDS
Fahnehjelm, [20]	ONH	NR^a^	–	–	FTF
Garcia-Filion, [21]	ONH	52/73 (71)	–	–	BDI
Groenveld, [23]	ONH	4/19 (21)	–	–	WISC-R, WPSSI, 10-item spatial ability scale
Margalith, [6]	ONH	36/51 (71)	–	–	Wechsler Test of Intelligence
Rivkees, [24]	ONH	8/19 (42)	–	–	BDI
Williams, [28]	ONH	1/7 (14)	1/7 (14) hyperactivity CBCL, psychosomatic CPRS	–	WPPSI-R, CBCL, CPRS^d^
Williams, [29]	ONH	–	–	3/7 (43) ASD^e^	ADOS, ADI-R, DSM-IV
Jutley-Neilson, * [32]	ONH ‘spectrum’		34/41 (83) sensory processing	–	SSP
Jutley-Neilson, [31]	SOD, ONH	NR	27/42 (64) mild-profound deficit VABS	23/42 (55) SCQ cut-off or clinical diagnosis	SIT-R3, VABS, SCQ
Parr, [33]	SOD, ONH	–	22/83 (27) > 1 SCRR	26/83 (31)	RZS, ICD-10
Griffiths, [22]	SOD	0/1 (0)	–	–	WISC
Severino, [18]	SOD	17/32 (53)	–	–	GDS, BSS
Vawter-Lee, [26]	SOD	0/2 (0)	–	–	ASQ-3
Webb, [27]	SOD	4/7 (57)	–	–	RZS
Alt, [19]	SOD, SOD-like, SOD-plus	11/16 (69)	–	*2/17 (12)*^*c*^	AIMS, BSID-II, DDST-II
Signorini, [25]	SOD, SOD-like, SOD-plus	8/17 (47)	–	–	RZS, GDS, WISC
Combined prevalence		175/336 (52%)	88/184 (48%)	65/187 (35%)	

Cognitive-developmental outcomes were measured by any formal intellectual, developmental, or cognitive testing

*–* indicates not assessed, *NR* Not reported, *CBCL* CHILD Behaviour Checklist, *FTF* Five to Fifteen Questionnaire, *WISC* Wechsler Intelligence Scale for Children, *WAIS* Wechsler Adult Intelligence Scale, *WPPSI* Wechsler Preschool and Primary Scale of Intelligence, *DSM* Diagnostic and Statistical Manual of Mental Disorders, *GDS* Griffiths Mental Development Scales, *NEPSY* Developmental Neuropsychological Assessment, *RZS* Reynell-Zinkin Scales, *ITVIC* Intelligence Test for Visually Impaired Children, *ADI-R* Autism Diagnostic Interview-Revised, *CARS* Childhood Autism Rating Scale-3, *BDI* Battelle Developmental Inventory, *CPRS* Conners Parent Rating Scale, *ADOS* Autism Diagnostic Observation Scale, *SSP* Short Sensory Profile, *SIT-R3* Slosson Intelligence Test-Revised, *VABS* Vineland Adaptive Behaviour Scale, *SCQ* Social Communication Questionnaire, *ICD-10* International Classification of Diseases-10, *BSS* Binet-Simon Scale, *ASQ-3* Ages and Stages Questionnaire-3, *AIMS* Alberta Infant Motor Scale, *BSID-II* Bayley Scales of Infant and Toddler Development-2, *DDST-II* Denver Developmental Screening Test-2

*Same sample as Jutley-Neilson [31]; unique results only reported

^a^Insufficient data available to report prevalence

^b^Two additional participants were diagnosed with autism-like condition (ALC) and one with ALC and oppositional defiant disorder

^c^No measurement given, therefore not included in prevalence estimate

^d^An extensive list of measures were used for different participants depending on visual difficulties; only example measures are listed

^e^Participants who received consistent diagnosis across all four measures included in prevalence

### Cognitive-developmental impairments

Intellectual disability or developmental problems were reported in 175 of 336 (52%) patients with available data (Table 2). One study [20] was not included in the grouped prevalence as insufficient information was reported to accurately estimate global developmental delay in their sample. Nevertheless, within their study, 51% of the sample displayed motor skill impairments and 43% presented perception problems [20]. One case study assessing specific spatial deficits in a 13-year-old girl with SOD and absent septum pellucidum found that she showed significant deficits in learning a floor maze and tracing lines (using kinaesthetic aids) compared to a blind-matched control [22]. Below, we discuss optic nerve involvement and neuroanatomical deficits as potential moderating factors in the association between SOD spectrum conditions and cognitive-developmental impairments.

Currently, the evidence regarding the association of optic nerve involvement or visual impairment and cognitive-developmental outcomes in SOD spectrum conditions is limited and mixed. Two studies [19, 25] observed a greater proportion of worsened developmental outcomes in participants with greater visual impairment. However, due to limited analyses, it is unclear how these findings may have been confounded by additional neuroanatomical or endocrine associations. Specifically, Signorini et al. [25] included a sample of predominantly bilateral ONH (16 of 17), and therefore, it is unknown how these results may be influenced by laterality of optic nerve involvement. Across three studies, it was consistently found that participants with bilateral ONH presented with greater impairments compared to those with unilateral ONH in relation to developmental dysfunction [20, 21] and intellectual disability [16]. These differences were likely contributed to, but not independently explained, by severity of visual impairment. Dahl et al. [16] found that the association between bilateral ONH and intellectual disability was the same for those with blindness to severe visual impairment (BCVA < 0.1) and those with moderate-to-mild visual impairment (BCVA > than 0.1) suggesting that visual impairment may not have contributed to intellectual disability and instead other clinical associations may have had greater effect. Similarly, Garcia-Filion et al. [21] found that worse visual acuity was associated with delay in adaptive and cognitive domains of the Battelle Developmental Inventory (BDI), but not overall developmental status, when adjusting for presence of corpus callosum hypoplasia. Therefore, while existing evidence suggests that bilateral (compared to unilateral) ONH is associated with greater cognitive-developmental impairment, it is uncertain yet if this association is linked with degree of visual impairment.

The presence of specific neuroanatomical and endocrine abnormalities have been associated with cognitive-developmental impairment in some, but not all, studies. Rivkees et al. [24] found a significantly higher prevalence of developmental delay in children with abnormal sleep–wake rhythmicity (100%) compared to those with normal rhythmicity (15%). Of those with sleep–wake rhythm disturbances, 66% had corpus callosum hypoplasia compared to 30% of those with normal sleep–wake rhythmicity [24]. Similarly, Garcia-Filion et al. [21] found that corpus callosum hypoplasia, but not absent septum pellucidum or pituitary gland malformations, was associated with increased developmental delay. In this study, it was also found that hypothyroidism was associated with delay in all domains of development, independent of corpus callosum hypoplasia [21]. Moreover, Severino et al. [18] reported a significant association between brain stem abnormalities and worsened developmental delay. Unexpectedly, Alt et al. [19] and Signorini et al. [25] found a high proportion of normal cognitive development in their participants diagnosed as SOD-plus (additional cortical malformations). Specifically, it was found that three of seven children with SOD-plus presented with normal cognitive development, while only two presented subnormal development and the other two borderline. Together, these findings suggest that specific neuroanatomical and endocrine deficits may be implicated in cognitive-developmental outcomes.

### Behavioural, emotional, and social impairments

Forty-eight percent (88 of 184) of children presented deficits within behavioural, emotional, or social domains (Table 2). Only three studies explored associations between the clinical features of SOD spectrum and neurobehavioural outcomes, and thus firm conclusions on the role of neuroanatomical, endocrine, and optic nerve involvement in the neurobehavioural phenotype remain to be clarified.

Two studies discussed how these neurobehavioural outcomes may have been moderated by visual impairment. Children with profound visual impairment were significantly more likely to present one of social communication, repetitive, or restrictive behavioural difficulties as well as presenting all three dysfunctions together, than children with severe visual impairment [33]. Jutley-Neilson et al. [32] similarly found a trend towards greater sensory processing difficulties in children with greater visual impairment. However, for both studies [32, 33], the distributions of additional neurological and hormone abnormalities across the visual impairment groups were unknown, and therefore may have confounded the pattern of results.

Controlling for visual loss, Webb et al. [15] recruited participants with no to mild-moderate visual impairment and normal cognition, and reported greater dysfunction on the anxious/depressed, withdrawn, thought problems, attention, aggressive, internalising, and externalising subscales of the CBCL, in addition to the global score, for the ONH group compared to age and sex-matched controls. Within the ONH group, four of 11 (36%) children had global CBCL scores within the range indicating presence of a clinical diagnosis. Importantly, the association of behavioural and emotional symptoms and ONH was independent of intellectual ability and visual impairment. It was also found that significantly reduced ventral cingulum fractional anisotropy (i.e. an index of white matter integrity) was associated with greater levels of behavioural problems [15]. Given that neurobehavioural impairments arise in the absence of visual loss, specific brain mechanisms may better explain the occurrence of these problems.

### Autism spectrum disorder

Autism spectrum disorder was measured using screening tools, validated questionnaires, and formal clinician diagnosis. Across five studies, 65 of 187 (35%) children presented a diagnosis of ASD or clinical level of symptoms (Table 2). Evidence for the aetiology underlying this prevalence, and the limitations of drawing firm conclusions, are discussed below.

Three studies assessed whether the prevalence of ASD symptoms depended on severity of visual impairment or optic nerve involvement. Parr et al. [33] found that the prevalence of ASD was numerically higher in children with profound visual impairment (36%) compared to those with severe visual impairment (27%), but this difference was not statistically significant. Jutley-Neilson et al. [31] found that children with greater visual loss had greater mean rank scores above the SCQ clinical cut-off for ASD than those with lesser visual impairment. However, it should be noted that the SCQ is not sensitive to the origin of abnormalities in social, communication, and restrictive behaviours in children with greater visual impairment, which may be specific adaptations following visual loss [31]. Dahl et al. [16] stratified ASD diagnosis in their sample by ONH type (i.e. bilateral or unilateral); five of 21 (24%) children with bilateral ONH and two of 21 (10%) children with unilateral ONH received a clinical diagnosis of ASD. This difference was not significant, and importantly, no association between visual acuity and ASD was reported in this sample [16]. Currently, the evidence regarding the association of ASD symptoms and visual impairment is mixed at best.

Ambiguity on the contribution of visual impairment to ASD may be partly limited by available measures. Williams et al. [29] compared ASD diagnoses across four methods of assessment: clinician, and adapted versions (for visual loss) of the Autism Diagnostic Observation Scale (ADOS) [34], the Autism Diagnostic Interview-Revised (ADI-R) [35], and ADI-R current behaviour (ADI-Rcb) subscale. Consistent diagnoses were only reached in three of seven children and the remaining four children had mixed diagnoses across each measure. One child was diagnosed with pervasive developmental disorder (PDD) by a clinician, autism by the ADOS, and no ASD by the ADI-R and ADI-Rcb [29]. The remaining three children were not diagnosed with ASD by the clinician, ADOS, or ADI-Rcb; however, received diagnoses of autism or PDD on the ADI-R [29]. Measures sensitive to visual impairment therefore remain to be developed and thus may limit conclusions on the prevalence and aetiology of ASD in these populations.

## Discussion

Neurodevelopmental impairments are known to be associated with SOD spectrum [5] and other conditions with lesions affecting the hypothalamic region. Yet, despite the formidable challenge they pose to the children and their families, neurodevelopmental impairments are not well-characterised and their prevalence has not been established. In this systematic review, we estimated that a high prevalence of 35 to 52% of children with SOD spectrum conditions may experience impairment across cognitive-developmental, behavioural, emotional, and social domains, or autism spectrum disorder. It should be noted that in order to accurately report on the prevalence of phenotypes such as ASD we urgently need clinical studies implementing formal diagnostic instruments with larger sample sizes. Additionally, we did not find sufficient evidence that visual impairment is independently associated with neurodevelopmental impairments, but we observed that it may instead be related to neuroanatomical or endocrine abnormalities.

Cognitive-developmental impairment has been the primary focus in research assessing children with SOD spectrum conditions, with the majority of studies assessing intellectual disability and developmental delay, rather than behavioural, social, or emotional outcomes. Notably, heterogeneity among the measures used to evaluate intellectual disability and developmental delay was observed. Additionally, we noted lack of consistency in the use of given measures with respect to the type of impairments they were reported to assess. For example, the Griffiths Mental Developmental Scale has been reported as a measure of developmental delay in one study [25], but also of intellectual disability in another study [30]. The heterogeneity and inconsistency in the use of measures may have been due to the wide age range and the variation in visual impairment across the samples. Similarly, the year of publication among the included studies ranged from 1984 to 2018, and therefore, authors may have been limited by measures available at the time. Nevertheless, we need to standardise assessments of intellectual disability and developmental functioning by age and degree of visual impairment using well-established measures to draw more valid comparisons across samples.

A number of studies did not meet the inclusion criteria of this review due to unclear definitions of outcomes, such as developmental delay, or for not reporting formal neurobehavioural assessments (see Additional File 2). While the included studies provided these formal assessments, the lack of validated measures of ASD for children with visual impairment [10] may have inflated our prevalence estimates due to misconstruing blind-specific behaviours for ASD symptoms in children with SOD spectrum conditions and visual impairment. It is plausible that blind-specific behaviours represent adaptations of the child due to visual loss as opposed to symptoms of ASD. Attempting to overcome this limitation, Williams et al. [29] modified the ADOS and ADI-R by removing or adapting vision related items to be appropriate for visually impaired children (e.g. for ADI-R ‘what does she/he do if someone else smiles at her/him’ to ‘what does she/he do if someone says something nice to her/him’). Despite these adjustments, consistent diagnosis across all four methods of assessment (i.e. clinician diagnosis, ADOS, ADI-R, ADI-Rcb) was only achieved in three of the seven participants in that study. This discrepancy suggests tools sensitive to visual impairment remain to be established in order to formulate a valid estimate of ASD and neurobehavioural dysfunction in children with varying levels of visual loss. In the absence of such instruments, repeated reviews of ASD symptoms over time may aid the delineation of blind-specific behaviours and neurodevelopmental disorder symptoms in visually impaired children [29]. For example, a child with visual impairment may develop adaptive social communication skills over time, whereas a child with ASD may continue to show communication and interaction delays throughout development. Additionally, future studies should focus on developing specific instruments that assess ASD based on non-visual features (as opposed to modifying existing measures) and ensure that these tools are validated in larger samples of children with varying severities of visual impairment [10].

### Clinical associations of neurodevelopmental impairments in septo-optic dysplasia

The mechanisms underlying neurodevelopmental impairments in children with septo-optic dysplasia are most likely multifactorial. Below, we explore the potential role of each of the core clinical features of SOD spectrum conditions, namely optic nerve involvement, neuroanatomical abnormalities, and the potential implications of endocrinological dysfunction.

It was consistently reported that children with bilateral ONH have greater intellectual disability [16] and developmental delay [20, 21] than those with unilateral ONH. Despite ONH presenting the primary cause of visual impairment across the SOD spectrum, the association between bilateral ONH and intellectual disability was found to be independent of visual acuity [16]. The clinical phenotype of bilateral ONH has been argued as more severe than unilateral ONH and is associated with greater risk of systemic abnormalities such as hypothalamic dysfunction and subsequent endocrine disturbance [21]. This suggests that additional neuroanatomical and endocrine abnormalities associated with bilateral ONH may contribute to intellectual deficits, as opposed to visual impairment.

The independent involvement of visual impairment in ASD symptomatology similarly remains to be elucidated. For example, two studies found that children with greater severity of visual impairment had greater dysfunctions across all social, communication, and repetitive and restrictive behavioural domains [31, 33]. While consistent with the argument that blind-specific abnormalities in social cognition may arise as a function of limited exposure to visual cues [11, 12], these studies did not account for the potential confounds of brain and endocrine abnormalities associated with the SOD condition. As a complex condition, it is unlikely that visual impairment solely accounts for the presentation of ASD symptomatology in SOD spectrum disorders.

At present, no study has assessed the relationship between brain abnormalities and ASD in SOD and only few have studied associations of brain abnormalities with neurodevelopmental impairments, highlighting an important area for future research. Of particular interest was the finding that greater behavioural dysfunction on the CBCL was associated with significantly reduced ventral cingulum fractional anisotropy in children with isolated ONH, no-to-moderate visual impairment, and no developmental delay [15]. Isolated ONH presents with no additional midline brain abnormalities or endocrine dysfunction. This finding calls for a more thorough characterisation of neuroanatomical deficits, even with apparently less-complex clinical phenotypes within the SOD spectrum, such as isolated ONH. It demonstrates that subtle white matter abnormalities in the brain may exist and that they are associated with the severity of behavioural deficits in this condition. Unexpectedly, it has also been reported that a high proportion of children with gross neuroanatomical abnormalities (SOD-plus) [4] were reported to have normal cognitive development [19, 25]. There may indeed be specific brain abnormalities that contribute to cognitive-developmental outcomes which requires further research and may inform which children within the SOD spectrum are at greater risk of neurobehavioural dysfunction and should be prioritised in care.

Hypothalamic neuropeptides, such as oxytocin and vasopressin, may be involved in neurodevelopmental impairments in conditions associated with hypothalamic-pituitary dysfunction [36]. For example, oxytocin is involved in the regulation of multiple physiological processes, with accumulating evidence for its role in the modulation of social behaviour and cognition [37]. A meta-analysis of 3941 participants found that three of 16 studied single-nucleotide polymorphisms of the *OXTR* gene were associated with ASD [38]. While no studies have characterised the role of oxytocin in SOD spectrum, experimental research has investigated the effects of administering intranasal oxytocin in children with ASD, conduct disorders, anxiety, and depression, reporting promising improvements in prosocial behaviours [39, 40]. Convergent evidence from functional neuroimaging research in children with ASD found that following administration of intranasal oxytocin compared to placebo, there was increased activity in the medial prefrontal cortex, superior temporal sulcus, and striatum in response to social stimuli, but not non-social stimuli [41]. Together, these findings suggest that oxytocin, may be implicated in dysfunctional social cognition and behaviour. Given the involvement of the hypothalamic-pituitary axis and prevalence of ASD in children with SOD spectrum, hypothalamic neuropeptides highlight a potential avenue for future investigation of neurodevelopmental impairments in this condition.

## Limitations

This systematic review aimed to assess the prevalence of neurodevelopmental impairments in children with SOD spectrum conditions. It must first be highlighted that this review could have been more inclusive due to genetic overlaps with other conditions (e.g. combined pituitary hormone deficiency) [42]. To maintain clarity in the definition of SOD spectrum condition, these conditions were not included. Inconsistencies in the diagnostic classification of SOD further highlighted the importance of considering SOD as a spectrum condition. Specifically, ONH was reported in a number of studies despite additional reports of endocrine and brain abnormalities, which would suggest a diagnosis of SOD. This may be partly due to the interchangeable use of ONH and SOD in earlier literature and the common progression of ONH into SOD in later childhood. Nevertheless, the available data from the studies included within this review suggests there may be a high prevalence of neurodevelopmental impairments in SOD spectrum conditions. High heterogeneity among the samples in relation to their neuroanatomical, endocrinological, and optic nerve involvement meant that it was not possible to statistically assess the relative contribution of these confounding factors. This was further limited by the variation in study designs and neurobehavioural assessments used, which may have increased the risk of information bias across the studies. This is particularly relevant to our review, given previous discussions of the limitations of assessing ASD behaviours in children with visual impairment [11].

Selection bias may also have arisen within the included studies, with parents supportive of an ASD diagnosis for their child potentially being more likely to engage with research assessing these outcomes. However, given that it has been argued that parents identify ASD-like traits in their children notably earlier than a diagnosis is reached [43], this may have strengthened the generalisability of the prevalence estimates from the included studies to the wider SOD spectrum population. Moreover, some study samples included a small number of participants older than 18 years; due to the rarity of the SOD condition, these studies were not excluded, and therefore, the age distribution should be considered when interpreting our findings.

## Conclusion

Overall, this review suggests that the prevalence of neurodevelopmental impairments in children within the SOD spectrum may be as high as 48 to 52%. Similarly, there is preliminary evidence that up to 35% of children with this condition may experience clinically relevant symptoms of ASD. The presence of hypothalamic dysfunction may pose a key shared mechanism underlying neurodevelopmental impairments in SOD spectrum conditions and other disorders with hypothalamic damage, such as Prader-Willi syndrome. However, drawing robust conclusions from the current literature remains challenging. Future research developing valid assessments of neurodevelopmental impairments may aid the delineation of blind-specific abnormalities in children with visual impairment compared to those with ASD and no visual impairment. Once valid measures for visually impaired children have been established, large multi-centre studies characterising the neurobehavioural phenotype of these children may inform future screening protocols for the ASD-like impairments in children with SOD spectrum. Nevertheless, at present, it is evident that children with SOD spectrum conditions experience neurodevelopmental impairments in need of intervention by appropriately trained clinicians, such as psychiatrists or clinical psychologists. Once established, clinicians should use screening tools modified for children with visual impairment alongside their specialist judgement in order to formulate a more valid assessment of neurodevelopmental impairments and ASD. Incorporating these assessments into routine care means diagnosis, which is required for therapeutic intervention by clinicians and educational support in schools, can be made for affected children. Moreover, since SOD spectrum conditions are diagnosed during the neonatal period, awareness that neurodevelopmental impairments are associated with the condition will support early diagnosis and therefore allow for intervention at an earlier developmental stage. In turn, this may improve the prognosis in children with SOD spectrum conditions, and subsequently minimise educational and behavioural challenges for patients and caregivers, and lead to improvements in their quality of life.

## Supplementary Information

**Additional file 1: Table S1.** Comparator group demographics and neurodevelopmental impairments for applicable studies.

**Additional file 2: Table S2.** Studies excluded due to not reporting validated measures of neurodevelopmental impairments.

## Data Availability

All data generated or analysed during this study are included in this published article [and its supplementary information files].

## Abbreviations

ADHD: Attention deficit hyperactivity disorder
ADI-R: Autism diagnostic interview-revised
ADI-Rcb: Autism diagnostic interview-revised current behaviour
ADOS: Autism diagnostic observation scale
ASD: Autism spectrum disorder
BCVA: Best corrected visual acuity
BDI: Battelle developmental inventory
CBCL: Child behaviour checklist
DSM-V: Diagnostic and statistical manual of mental health disorders: 5th edition
ONH: Optic nerve hypoplasia
PDD: Pervasive developmental disorder-not otherwise specified
PRISMA: Preferred reporting items for systematic reviews and meta-analyses
SCQ: Social communication questionnaire
SOD: Septo-optic dysplasia
SOD-plus: Septo-optic dysplasia plus
