# Supplementary material for: Neurodevelopmental impairments in children with septo-optic dysplasia spectrum conditions: a systematic review
Source: Mol Autism. 2023 Jul 25;14:26. doi: 10.1186/s13229-023-00559-0 (PMC10369759; doi:10.1186/s13229-023-00559-0)
Supplement: Supplementary file 1 — Additional file 1: Table S1. Comparator group demographics and neurodevelopmental impairments for applicable studies. [file 13229_2023_559_MOESM1_ESM.pdf]

Additional File 1

Table S1. Comparator group demographics and neurodevelopmental impairments for applicable studies.

First Author, Year	SOD Spectrum Group		Comparator Group			Group Differences
	Sample Size (N)	Neurodevelopmental impairments	Sample size (N)	Demographics	Neurodevelopmental impairments	
Webb, 2013	11	Mean CBCL Total Score = 63.6	11	Typically developing age- and sex-matched controls	Mean CBCL Total Score = 51.3	No difference in FSIQ, VIQ, PIQ (all p >0.05)<br><br>ONH greater behavioural problems than controls (p= 0.006)
Fahnehjelm, 2003	28	9/24 IQ <70<br>8/24 ASD	12	Coloboma/ microphthalmos*	5/10 IQ <70	No difference in IQ (p > 0.05)
Groenveld, 1994	19	4/19 VIQ <70	118	Visually impaired, no neurological abnormalities controls *	NR	NR
Griffiths, 1984	1	VIQ = 122<br><br>FM = 21<br>KT = 27<br>AC = 13	1	Blind male, 11.7 years, Leber's congenital amaurosis	VIQ = 114<br><br>FM = 87<br>KT = 70<br>AC = 11	Control percentage correct on FM and KT (p=0.01). No difference on AC
Severino, 2014	38	17/32 developmental delay	114	Healthy age- and sex- matched controls	Normal developmental profile	NR

AC indicates Angle Copying Test mean discrepancy; CBCL, Child Behaviour Checklist; FM, Floor Maze Test percentage correct; FSIQ, Full Scale Intelligence Quotient; IQ, Intelligence Quotient; KT, Kinaesthetic Tracing Test percentage correct; NR, not reported; PIQ, Performance Intelligence Quotient; VIQ, Verbal Intelligence Quotient. *Age and sex not reported for sample.

References

Fahnehjelm, K. T., Wide, K., Flodmark, O., Ek, U., & Hellström, A. (2003). Posterior ocular malformations in children: somatic, neuroradiological and cognitive aspects. *Acta Paediatrica*, *92*(3), 301-308.

Griffiths, P., & Hunt, S. (1984). Specific spatial defect in a child with septo-optic dysplasia. *Developmental Medicine & Child Neurology*, *26*(3), 395-400.

Groenveld, M., Pohl, K. R., Espezel, H., & Jan, J. E. (1994). The septum pellucidum and spatial ability of children with optic nerve hypoplasia. *Developmental Medicine & Child Neurology*, *36*(3), 191-197.

Severino, M., Allegri, A. E. M., Pistorio, A., Roviglione, B., Di Iorgi, N., Maghnie, M., & Rossi, A. (2014). Midbrain-hindbrain involvement in septo-optic dysplasia. *American Journal of Neuroradiology*, *35*(8), 1586-1592.

Webb, E. A., O'Reilly, M. A., Clayden, J. D., Seunarine, K. K., Dale, N., Salt, A., ... & Dattani, M. T. (2013). Reduced ventral cingulum integrity and increased behavioral problems in children with isolated optic nerve hypoplasia and mild to moderate or no visual impairment. *Plos One*, *8*(3), e59048.
