# Supplementary material for: Neurodevelopmental impairments in children with septo-optic dysplasia spectrum conditions: a systematic review
Source: Mol Autism. 2023 Jul 25;14:26. doi: 10.1186/s13229-023-00559-0 (PMC10369759; doi:10.1186/s13229-023-00559-0)
Supplement: Supplementary file 2 — Additional file 2: Table S2. Studies excluded due to not reporting validated measures of neurodevelopmental impairments. [file 13229_2023_559_MOESM2_ESM.pdf]

Additional File 2

Table S2. Studies excluded due to not reporting validated measures of neurodevelopmental impairments.

Title	First Author, Year	Exclusion Justification
A Case of Congenital Hypopituitarism Associated With a 1p31 Microdeletion: A Possible Role for LEPR and JAK1.	Thakur, 2017	Mentions behavioural problems – no validated measure reported.
Antenatal diagnosis of absence of septum pellucidum.	M'Barek, 2020	Mentions language delay- but no validated measures reported.
Children with blindness - major causes, developmental outcomes and implications for habilitation and educational support: a two-decade, Swedish population-based study.	de Verdier, 2018	Clinical records reviewed for cognitive level, neurodevelopmental impairments and type of school placement – no validated measure reported.
Children with septo-optic dysplasia - how to improve and sharpen the diagnosis.	Hellström, 2000	Based on clinical records – no validated measures reported.
Congenital Blindness and Autism Spectrum Disorder	Conrad, 2021	Commentary style case report – no validated measures reported.
Demographic, ocular and associated neurological findings in corpus callosum malformations	Kiziltunc, 2021	No validated measures reported
Facilitation of drug-resistant epilepsy and catastrophic status epilepticus in children with combined pituitary hormone deficiency	Schönberger, 2021	Based on clinical records – no validated measures reported.
Increasing incidence of optic nerve hypoplasia/septo-optic dysplasia spectrum: Geographic clustering in Northern Canada.	Khaper, 2017	Retrospective chart review - no validated measures reported
Optic nerve hypoplasia in children. Association with anomalies of the endocrine and CNS.	Skarf, 1984	Discusses developmental delay, but this is not defined – no validated measures reported

Optic nerve hypoplasia with hypopituitarism. Septo-optic dysplasia with hypopituitarism.	Patel, 1975	Discusses delayed behavioural development - no validated measures reported
Optic nerve hypoplasia. Clinical significance of associated central nervous system abnormalities on magnetic resonance imaging.	Brodsky, 1993	No validated measures reported- developmental delay listed in table, not indicated as an outcome of the study
Self-aggression and congenital clubfoot: Additional features to the septo-optic dysplasia complex	Peruchi, 2009	Clinician report/ observations – no validated measures reported
Spectrum of clinical presentations and endocrinological findings of patients with septo-optic dysplasia: A retrospective study	Cemeroglu, 2015	Clinical records used for behavioural, autism, and cognitive results- no validated measures reported.
The central diabetes insipidus associated with septo-optic dysplasia (de Morsier syndrome).	Hetman, 2018	Psychomotor developmental delay stated- no validated measures reported
Wide cavum septum pellucidum: a marker of disturbed brain development.	Bodensteiner, 1990	States that all patients had some assessment of intellectual function which consisted of formal neuropsychologic testing or developmental assessment, but measure not reported.
Agenesis of the septum pellucidum: Prenatal diagnosis and outcome	Borkowski-Tillman, 2020	Developmental delay reported in table - no validated measures reported.
Brain and ocular abnormalities in infants with in utero exposure to cocaine and other street drugs	Dominguez, 1991	Measures reported, but unclear which measures were used for SOD participants in sample.
Cerebral midline developmental anomalies: endocrine, neuroradiographic and ophthalmological features	Antonini, 2002	Psychomotor retardation reported - no validated measures reported.
Clinical and demographic associations with optic nerve hypoplasia in New Zealand	Goh, 2014	Developmental delay assessed based on developmental milestones, but no validated measure reported.
Clinical characteristics of septo-optic dysplasia accompanied by congenital central hypothyroidism in Japan	Nagasaki, 2017	Developmental delay reported in table – no validated measures reported.

De Morsier syndrome associated with periventricular nodular heterotopia: case report	Spinosa, 2007	Mentions delay in language, motor developmental milestones – no validated measure reported.
Fetal Ultrasound and Magnetic Resonance Imaging Findings in Suspected Septo-Optic Dysplasia: A Diagnostic Dilemma	Maduram, 2020	Neurodevelopmental delays reported from clinical notes – no validated measure reported.
Hypopituitarism and neurodevelopmental abnormalities in relation to central nervous system structural defects in children with optic nerve hypoplasia	Haddad, 2005	Developmental deficits and behavioural problems reported. Questionnaire used, but measured development via school performance or if in SEN school – no validated measures reported.
Hypoplastic optic nerves and pituitary dysfunction. A spectrum of anatomical and endocrine abnormalities	Stanhope, 1984	Developmental delay mentioned – no validated measures reported.
Is there an encephalographic trait to septo-optic dysplasia? (de Morsier syndrome)	Tarta-Arsene, 2014	No validated measure reported - one case suspected SOD and developmental delays
Long-term postnatal outcome of fetuses with prenatally suspected septo-optic dysplasia	Shinar, 2020	'Standardised developmental assessment' used but not defined in terms of outcomes – no validated measure reported.
Mutations in LAMB2 Are Associated With Albuminuria and Optic Nerve Hypoplasia With Hypopituitarism	Tahoun, 2020	Reports global developmental delay but no validated measure reported.
Nailing septo-optic dysplasia	Palui, 2018	Delayed developmental milestones reported – no validated measure reported.
Neuroimaging and endocrine disorders in paediatric optic nerve hypoplasia	Qian, 2018	Developmental delay data extracted from medical records – no validated measure reported.
Ocular, neurologic and systemic findings of the cases with optic nerve hypoplasia	Karahan, 2016	Neurodevelopmental outcomes from medical records - no validated measures reported.
Optic nerve hypoplasia associated with absent septum pellucidum and hypopituitarism	Krause-Brucker, 1980	Delayed developmental milestones reported – no validated measures reported.
Optic nerve hypoplasia, encephalopathy, and neurodevelopmental handicap	Burke, 1991	Neurodevelopmental handicap discussed as mental retardation, cerebral palsy, and epilepsy – no validated measure reported.

Pituitary dysfunction, morbidity and mortality with congenital midline malformation of the cerebrum	Cameron, 1999	Neurodevelopmental disability from records – no validated measures reported.
Refining clinical phenotypes in septo-optic dysplasia based on MRI findings	Riedl, 2008	Developmental evaluation based on school type/ school performance – no validated measures or clear outcome reported.
Schizencephaly in children: Clinical features and associated findings	Wongnate, 2020	Developmental disability defined by presence of abnormal mental neurodevelopment and language abnormalities – but no validated measures reported.
Septo-optic dysplasia (case report)	Nayak, 1991	Normal developmental milestones reported – no validated measures reported.
Septo-optic dysplasia [SOD] and endocrine abnormalities in Khon Kaen, Thailand	Sangkha, 2018	Developmental delay reported but no indication of measurement
Septo-optic dysplasia plus: a patient with diabetes insipidus	Carman, 2010	Developmental delay reported – no validated measures reported.
Septo-optic dysplasia plus: a spectrum of malformations of cortical development	Miller, 2000	Global developmental delay reported – no validated measures reported.
Septo-optic dysplasia with digital anomalies	Pagon, 1984	Measurement of development/ intellectual ability not clearly defined.
Unilateral optic nerve hypoplasia with asymmetric septum: A case report of unilateral septo-optic dysplasia	Riviello, 2014	Developmental milestones reported to be met – but no validated measures reported.

References

Antonini, S. R., Grecco Filho, A., Elias, L. L., Moreira, A. C., & Castro, M. (2002). Cerebral midline developmental anomalies: endocrine, neuroradiographic and ophthalmological features. *Journal of Pediatric Endocrinology & Metabolism,15*(9), 1525–1530. https://doi.org/10.1515/jpem.2002.15.9.1525

Bodensteiner, J. B., & Schaefer, G. B. (1990). Wide cavum septum pellucidum: a marker of disturbed brain development. *Pediatric Neurology*, *6*(6), 391–394. https://doi.org/10.1016/0887-8994(90)90007-n

Borkowski-Tillman, T., Garcia-Rodriguez, R., Viñals, F., Branco, M., Kradjen-Haratz, K., Ben-Sira, L., Lerman-Sagie, T., & Malinger, G. (2020). Agenesis of the septum pellucidum: Prenatal diagnosis and outcome. *Prenatal Diagnosis*, *40*(6), 674–680. https://doi.org/10.1002/pd.5663

Brodsky, M. C., & Glasier, C. M. (1993). Optic nerve hypoplasia. Clinical significance of associated central nervous system abnormalities on magnetic resonance imaging. *Archives of Ophthalmology (Chicago, Ill.:1960)*, *111*(1), 66–74. https://doi.org/10.1001/archopht.1993.01090010070029

Burke, J. P., O'Keefe, M., & Bowell, R. (1991). Optic nerve hypoplasia, encephalopathy, and neurodevelopmental handicap. *The British Journal of Ophthalmology*, *75*(4), 236–239. https://doi.org/10.1136/bjo.75.4.236

Cameron, F. J., Khadilkar, V. V., & Stanhope, R. (1999). Pituitary dysfunction, morbidity and mortality with congenital midline malformation of the cerebrum. *European Journal of Pediatrics*, *158*(2), 97–102. https://doi.org/10.1007/s004310051026

Carman, K. B., Yarar, C., Yakut, A., & Adapinar, B. (2010). Septo-optic dysplasia plus: a patient with diabetes insipidus. *Pediatric Neurology*, *43*(1), 76–78. https://doi.org/10.1016/j.pediatrneurol.2010.03.001

Cemeroglu, A. P., Coulas, T., & Kleis, L. (2015). Spectrum of clinical presentations and endocrinological findings of patients with septo-optic dysplasia: a retrospective study. *Journal of Pediatric Endocrinology & Metabolism, 28*(9-10), 1057–1063. https://doi.org/10.1515/jpem-2015-0008

Conrad, C., Linnea, K., & Augustyn, M. (2021). Congenital Blindness and Autism Spectrum Disorder. *Journal of Developmental and Behavioral Pediatrics, 42*(2), 163–165. https://doi.org/10.1097/DBP.0000000000000905

de Verdier, K., Ulla, E., Löfgren, S., & Fernell, E. (2018). Children with blindness – major causes, developmental outcomes and implications for habilitation and educational support: a two-decade, Swedish population-based study. *Acta Ophthalmologica*, *96*(3), 295–300. https://doi.org/10.1111/aos.13631

Dominguez, R., Aguirre Vila-Coro, A., Slopis, J. M., & Bohan, T. P. (1991). Brain and ocular abnormalities in infants with in utero exposure to cocaine and other street drugs. *American Journal of Diseases of Children (1960)*, *145*(6), 688–695. https://doi.org/10.1001/archpedi.1991.02160060106030

Goh, Y. W., Andrew, D., McGhee, C., & Dai, S. (2014). Clinical and demographic associations with optic nerve hypoplasia in New Zealand. *The British Journal of Ophthalmology*, *98*(10), 1364–1367. https://doi.org/10.1136/bjophthalmol-2013-304605

Haddad, N. G., & Eugster, E. A. (2005). Hypopituitarism and neurodevelopmental abnormalities in relation to central nervous system structural defects in children with optic nerve hypoplasia. *Journal of Pediatric Endocrinology & Metabolism*, *18*(9), 853–858. https://doi.org/10.1515/jpem.2005.18.9.853

Hellström, A., Aronsson, M., Axelson, C., Kyllerman, M., Kopp, S., Steffenburg, S., Strömland, K., Westphal, O., Wiklund, L., & Albertsson Wikland, K. (2000). Children with septo-optic dysplasia - how to improve and sharpen the diagnosis. *Hormone Research*, *53, S1*, 19–25. https://doi.org/10.1159/000053200

Hetman, M., Fułek, M., Zajączkowska, K., Żarczyńska, A., Łagosz, P., & Barg, E. (2018). The central diabetes insipidus associated with septo-optic dysplasia (de Morsier syndrome). *Pediatric Endocrinology, Diabetes, and Metabolism*, *24*(4), 197–203. https://doi.org/10.5114/pedm.2018.83367

Karahan, E., & Tulin Berk, A. (2016). Ocular, neurologic and systemic findings of the cases with optic nerve hypoplasia. *The Open Ophthalmology Journal*, *10*, 5–11. https://doi.org/10.2174/1874364101610010005

Khaper, T., Bunge, M., Clark, I., Rafay, M. F., Mhanni, A., Kirouac, N., Sharma, A., Rodd, C., & Wicklow, B. (2017). Increasing incidence of optic nerve hypoplasia/septo-optic dysplasia spectrum: Geographic clustering in Northern Canada. *Paediatrics & Child Health*, *22*(8), 445–453. https://doi.org/10.1093/pch/pxx118

Kızıltunç, P. B., Şahlı, E., İdil, A., & Atilla, H. (2021). Demographic, ocular and associated neurological findings in corpus callosum malformations. *The Turkish Journal of Pediatrics*, *63*(2), 291–299. https://doi.org/10.24953/turkjped.2021.02.013

Krause-Brucker, W., & Gardner, D. W. (1980). Optic nerve hypoplasia associated with absent septum pellucidum and hypopituitarism. *American Journal of Ophthalmology*, *89*(1), 113–120. https://doi.org/10.1016/0002-9394(80)90237-8

Lin, J., Peruchi, M.M., Masruha, M.R., Pacheco, J.P., & Vilanova, L.C. (2009). Self-aggression and congenital clubfoot: additional features to the septo-optic dysplasia complex. *Arquivos de Neuro-Psiquiatria, 67 2A*, 299-301 .

Maduram, A., Farid, N., Rakow-Penner, R., Ghassemi, N., Khanna, P. C., Robbins, S. L., Hull, A., Gold, J., & Pretorius, D. H. (2020). Fetal ultrasound and magnetic resonance imaging findings in suspected septo-optic dysplasia: A diagnostic dilemma. *Journal of Ultrasound in Medicine: Official Journal of the American Institute of Ultrasound in Medicine*, *39*(8), 1601–1614. https://doi.org/10.1002/jum.15252

Miller, S. P., Shevell, M. I., Patenaude, Y., Poulin, C., & O'Gorman, A. M. (2000). Septo optic dysplasia plus: a spectrum of malformations of cortical development. *Neurology*, *54*(8), 1701–1703. https://doi.org/10.1212/wnl.54.8.1701

M'Barek, B. I., Tassin, M., Guët, A., Simon, I., Mairovitz, V., Mandelbrot, L., & Picone, O. (2020). Antenatal diagnosis of absence of septum pellucidum. *Clinical Case Reports*, *8*(3), 498–503. https://doi.org/10.1002/ccr3.2666

Nagasaki, K., Kubota, T., Kobayashi, H., Sawada, H., Numakura, C., Harada, S., Takasawa, K., Minamitani, K., Ishii, T., Okada, S., Kamasaki, H., Sugihara, S., Adachi, M., & Tajima, T. (2017). Clinical characteristics of septo-optic dysplasia accompanied by congenital central hypothyroidism in Japan. *Clinical Pediatric Endocrinology: Case Reports and Clinical Investigations: Official Journal of the Japanese Society for Pediatric Endocrinology*, *26*(4), 207–213. https://doi.org/10.1297/cpe.26.207

Nayak, V., & Bhat, K. R. (1991). Septo-optic dysplasia (case report). *Indian Journal of Ophthalmology*, *39*(4), 186–187.

Pagon, R. A., & Stephan, M. J. (1984). Septo-optic dysplasia with digital anomalies. *The Journal of Pediatrics*, *105*(6), 966–968. https://doi.org/10.1016/s0022-3476(84)80092-x

Palui, R., Sahoo, J. P., Kamalanathan, S., & Sridharan, K. (2018). Nailing septo-optic dysplasia. *BMJ Case Reports*, *2018*, 2017223956. https://doi.org/10.1136/bcr-2017-223956

Patel, H., Tze, W. J., Crichton, J. U., McCormick, A. Q., Robinson, G. C., & Dolman, C. L. (1975). Optic nerve hypoplasia with hypopituitarism. Septo-optic dysplasia with hypopituitarism. *American Journal of Diseases of Children (1960)*, *129*(2), 175–180. https://doi.org/10.1001/archpedi.1975.02120390017004

Qian, X., Fouzdar Jain, S., Morgan, L. A., Kruse, T., Cabrera, M., & Suh, D. W. (2018). Neuroimaging and endocrine disorders in paediatric optic nerve hypoplasia. *The British Journal of Ophthalmology*, *102*(7), 906–910. https://doi.org/10.1136/bjophthalmol-2017-310763

Riedl, S., Vosahlo, J., Battelino, T., Stirn-Kranjc, B., Brugger, P. C., Prayer, D., Müllner Eidenböck, A., Kapelari, K., Blümel, P., Waldhör, T., Krasny, J., Lebl, J., & Frisch, H. (2008). Refining clinical phenotypes in septo-optic dysplasia based on MRI findings. *European Journal of Pediatrics*, *167*(11), 1269–1276. https://doi.org/10.1007/s00431-007-0666-x

Riviello, P. J., Tyagi, V., & Milla, S. S. (2014). Unilateral optic nerve hypoplasia with asymmetric septum: A case report of unilateral septo-optic dysplasia. *Journal of Pediatric Neuroradiology, 3,* 75-79.

Sangkha, N., Wiromrat, P., Panamonta, M., & Panamonta, O. (2018). Septo-optic dysplasia (SOD) and endocrine abnormalities in Khon Kaen, Thailand. *Journal of the Medical Association of Thailand*, *101*(5)*,* 71.

Schönberger, J., Eckenweiler, M., Klotz, K. A., Sag, Y., Shah, M., Fuchs, H., Kirschner, J., & Jacobs, J. (2021). Facilitation of drug-resistant epilepsy and catastrophic status epilepticus in children with combined pituitary hormone deficiency. *European Journal of Paediatric Neurology: Official Journal of the European Paediatric Neurology Society*, *33*, 99–105. https://doi.org/10.1016/j.ejpn.2021.05.016

Shinar, S., Blaser, S., Chitayat, D., Selvanathan, T., Chau, V., Shannon, P., Agrawal, S., Ryan, G., Pruthi, V., Miller, S. P., Krishnan, P., & Van Mieghem, T. (2020). Long term postnatal outcome of fetuses with prenatally suspected septo-optic dysplasia. *Ultrasound in obstetrics & Gynecology: The Official Journal of the International Society of Ultrasound in Obstetrics and Gynecology*, *56*(3), 371–377. https://doi.org/10.1002/uog.22018

Skarf, B., & Hoyt, C. S. (1984). Optic nerve hypoplasia in children. Association with anomalies of the endocrine and CNS. *Archives of ophthalmology (Chicago, Ill. : 1960)*, *102*(1), 62–67. https://doi.org/10.1001/archopht.1984.01040030046032

Spinosa, M. J., Liberalesso, P. B., Vieira, S. C., & Löhr, A., Jr (2007). De Morsier syndrome associated with periventricular nodular heterotopia: case report. *Arquivos de Neuro Psiquiatria*, *65*(3A), 693–696. https://doi.org/10.1590/s0004-282x2007000400029

Stanhope, R., Preece, M. A., & Brook, C. G. (1984). Hypoplastic optic nerves and pituitary dysfunction. A spectrum of anatomical and endocrine abnormalities. *Archives of Disease in Childhood*, *59*(2), 111–114. https://doi.org/10.1136/adc.59.2.111

Tahoun, M., Chandler, J. C., Ashton, E., Haston, S., Hannan, A., Kim, J. S., D'Arco, F., Bockenhauer, D., Anderson, G., Lin, M. H., Marzouk, S., Saied, M. H., Miner, J. H., Dattani, M. T., & Waters, A. M. (2020). Mutations in LAMB2 Are Associated With Albuminuria and Optic Nerve Hypoplasia With Hypopituitarism. *The Journal of Clinical Endocrinology and Metabolism*, *105*(3), 595–599. https://doi.org/10.1210/clinem/dgz216

Tarta-Arsene, O., Leanca, M., Gander, M., & Craig, D. (2014). Is there an encephalographic trait to septo-optic dysplasia? (de Morsier syndrome). *Romanian Journal of Neurology, 8*(2), 75-80.

Thakur, M., Taha, D., & Misra, V. K. (2017). A Case of Congenital Hypopituitarism Associated With a 1p31 Microdeletion: A Possible Role for *LEPR* and *JAK1*. *Journal of the Endocrine Society*, *1*(4), 278–282. https://doi.org/10.1210/js.2016-1072

Wongnate, J., Danchaivijitr, N., & Likasitwattanakul S. (2020). Schizencephaly in children: Clinical features and associated findings. *Journal of the Medical Association of Thailand, 103*, 14-21.
